# Brain Plasticity in Mammals: An Example for the Role of Comparative Medicine in the Neurosciences

**DOI:** 10.3389/fvets.2018.00274

**Published:** 2018-11-01

**Authors:** Chiara La Rosa, Luca Bonfanti

**Affiliations:** ^1^Neuroscience Institute Cavalieri Ottolenghi, Turin, Italy; ^2^Department of Veterinary Sciences, University of Turin, Turin, Italy

**Keywords:** comparative studies, sheep, immature neurons, translation, cerebral cortex, amygdala, comparative neuroanatomy

## Abstract

Comparative medicine deals with similarities and differences between veterinary and human medicine. All mammals share most basic cellular and molecular mechanisms, thus justifying murine animal models in a translational perspective; yet “mice are not men,” thus some biases can emerge when complex biological processes are concerned. Brain plasticity is a cutting-edge, expanding topic in the field of Neurosciences with important translational implications, yet, with remarkable differences among mammals, as emerging from comparative studies. In particular, adult neurogenesis (the genesis of new neurons from brain stem cell niches) is a life-long process in laboratory rodents but a vestigial, mostly postnatal remnant in humans and dolphins. Another form of “whole cell” plasticity consisting of a population of “immature” neurons which are generated prenatally but continue to express markers of immaturity during adulthood has gained interest more recently, as a reservoir of young neurons in the adult brain. The distribution of the immature neurons also seems quite heterogeneous among different animal species, being confined within the paleocortex in rodents while extending into neocortex in other mammals. A recent study carried out in sheep, definitely showed that gyrencephalic, large-sized brains do host higher amounts of immature neurons, also involving subcortical, white, and gray matter regions. Hence, “whole cell” plasticity such as adult neurogenesis and immature neurons are biological processes which, as a whole, cannot be studied exclusively in laboratory rodents, but require investigation in comparative medicine, involving large-sized, long-living mammals, in order to gain insights for translational purposes.

## Introduction

Comparative medicine, namely, “the field of study concentrating on similarities and differences between veterinary and human medicine,” relies on the fact that “mice are not men” and thus laboratory rodents cannot always be the best models for human physiology and disease (1). Though mice and humans share striking biological similarities, mainly regarding basic molecular mechanisms which can be profitably explored by genetic manipulation, important differences and biases also emerge when complex biological processes are concerned. Dominant models may bias research directions or omit important context, possibly hampering reliable translational outcomes (2). In some cases, other animal models are required and the experience/expertise of the veterinary sciences might be involved. Nevertheless, if veterinary and human medicine are steeped in a rich history of “One Medicine,” they have paradoxically parted ways, still not recognizing that they speak different dialects of the same language (1, 3). As stated by Schwabe (4), “the attempt to biologically pseudo-oligarchy-ize the human among species is one of the most pervasive mistakes of modern science,” and this is a pity for veterinary research, which seems to be intended to always remain in the background, not fully achieving the One Health purpose. The success of the One Health concept requires breaking down the interdisciplinary barriers that still separate human and veterinary medicine, but also ecological, evolutionary and environmental sciences (5). In this context, we strongly feel that many hidden opportunities do exist to develop engaging and useful themes falling in the landscape of the veterinary field of interest and being necessary to human health, not only referring to comparative pathology but also involving basic sciences. One of the most investigated topics in modern Neurosciences is neural plasticity, namely, the capability of the brain to change its structure/function throughout life in response to environmental challenges, thus being fundamental for species/individual adaptation and, notably, for brain aging and repair (6, 7). Due to its different features/types in relation to different neuroanatomies, environments and animal behavior, neural plasticity is a typical example of a complex biological process substantially differing among mammalian species (8, 9). In this perspective article we will address the reasons making brain plasticity an excellent topic for comparative medicine, also illustrating the current and future chances for developing it.

## Brain plasticity in mammals: why is it important?

**Figure 1:**
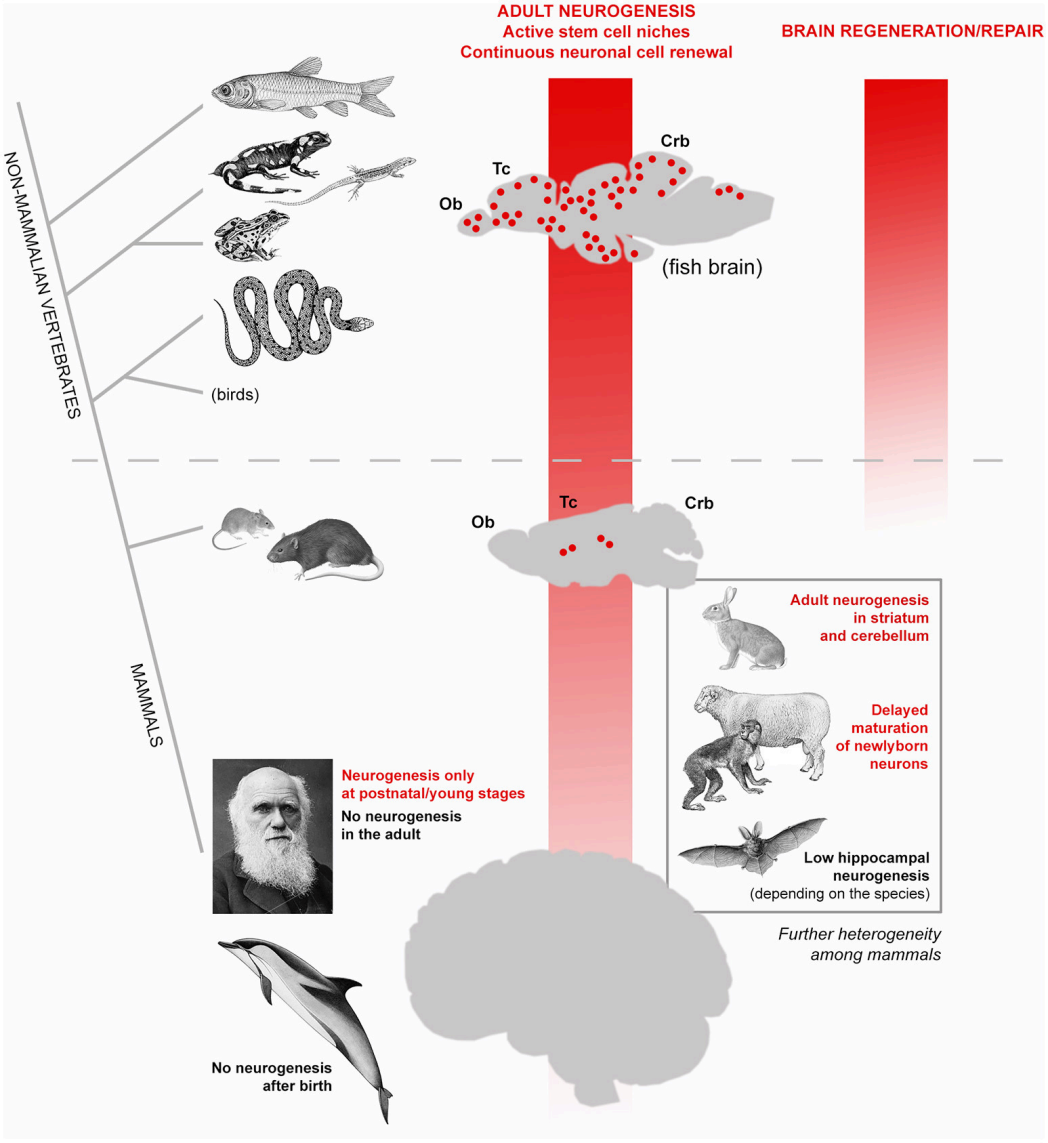
High heterogeneity of adult neurogenesis occurrence and role(s) across vertebrates and, to a lesser extent, among mammals. Vertical red shades indicate general trends of progressive reduction of adult neurogenesis from constitutive stem cell niches (left) and its regenerative/repair capacity (right). From fish to man, a dramatic shift occurs: from widely distributed neurogenic zones granting continuous cell renewal in most brain regions, also allowing lesion-induced regeneration (see text), to a substantially static brain tissue, in which addition of new neurons is mostly granted at postnatal/young stages and cannot efficiently renew/repair itself. In this general trend, remarkable differences also exist among mammalian species: laboratory rodents still have constitutive neurogenesis throughout life whereas in humans no active stem cell niches are detectable in adulthood. The evolutionary rules behind the general trend of reduction are still obscure but it is clear that such process is not linear, rather made more complex by a high heterogeneity revealed by comparative studies carried out in non-rodent mammals (bottom right).

In modern societies, neurological diseases, dementia and other age-related problems affecting the human brain represent a heavy health, social and economic burden (10). The progressive extension of human lifespan expectancy (11) will produce increasing numbers of individuals affected by neurological problems worldwide, thus making the issue of age-related dementia a global priority (12). Despite intensive research in Neuroscience, most brain pathologies involving progressive degeneration or functional loss of neurons still fall into the category of incurable diseases. Such failure is strictly linked to a well-known feature of the mammalian nervous tissue: its scarce capacity of renewing the cellular components through adulthood (most neurons are lifelong elements) and to undergo efficacious reparative processes after damage (7, 13, 14). In other words, despite thousands of papers published on the mechanisms of neural plasticity, our brain seems to suffer a substantial lack of structural plasticity. An explanation can be found in comparative medicine. Neurobiological research carried out on non-mammalian vertebrates clearly shows that fish, amphibians and reptiles do renew most neuronal populations throughout life and perform regeneration/repair after lesion (14, 15) (Figure 1). By contrast, both cell renewal and efficacious regenerative processes are not a natural property of the mammalian nervous system, likely due to evolutionary constraints involving a combination of factors [among them: brain complexity, scarcity of stem cells, incapability of cell de-differentiation, role of immune system; (7, 13, 16). As a result, apart from widespread synaptic plasticity, which operates on the pre-existing neuronal elements and only in very small portions of the cell [dendritic spines, apical part of axons; see (17)], a “whole cell” plasticity involving changes in the overall shape and/or number of cells is a rare event. Usually, in mammals, the genesis of new neurons (adult neurogenesis) is highly spatially-restricted to two stem cell niches (the forebrain subventricular zone and the subgranular zone of the hippocampus; Figure 1). A third neurogenic zone is known to exist in the rodent hypothalamus, and other small spots of cell proliferation have been described to occur in other regions, yet none of them leading to fully structural/functional integration of the newlyborn neurons [reviewed in (18, 19)]. During the last three decades, most efforts of Neurodevelopmental research have been directed to foster residual “whole cell” plasticity in the mammalian nervous system, with the aim of reactivating intrinsic/silent programs of cell genesis/regeneration. Some cellular reactions can be experimentally-induced after lesion in murine models. For instance, activation of stem/progenitor cells in the neurogenic niches (20, 21) or local activation of neurogenic astrocytes (22, 23), can be elicited by inflammation/cell death and are followed by migration of neuroblasts toward the site of lesion. Though reminiscent of the first phases of reparative processes occurring in non-mammalian vertebrates (24), most of these tissue reactions ultimately do not lead to brain repair and cell replacement, as described in the widely plastic fish brain (reviewed in 15). Instead, they are followed by disappearance of the newlyborn cells, which have a transient existence (21, 23) during which they can exert “bystander” effects through the secretion of molecules/microvescicles in the site of lesion [reviewed in (7, 25)]. In parallel, different types of stem/progenitor cells have been harvested, cultured or produced *in vitro* (e.g., induced pluripotent stem cells) to be used for transplantation. Yet, no substantial efficacious treatments have been achieved in clinical trials for neurodegenerative diseases carried out in humans and based on stem cell treatments (26, 27). Out of 300 trials started before 2012 in humans, no one led to efficacious treatment and final approval (28), what consists of high costs in terms of money and time. Even the most prominent scientists working on these issues and trying to use plasticity to foster brain repair agree that it would be premature to launch clinical trials to use stem cells to treat neurological disorders and that further preclinical studies are needed (26, 27). On the whole, such efforts still clash against the above-mentioned evolutionary constraints, not taking into account an aspect which was underestimated since the beginning: the remarkable differences existing among mammalian species.

## Differences in brain plasticity among mammals

Since their discovery in the nineties (29, 30), adult neural stem cells and their continuous renewal of neurons in the olfactory bulb and hippocampal dentate gyrus of rodents were intensively studied. Such research was fueled by the hope of using plasticity to replace lost/damaged neurons (both from endogenous—constitutive adult neurogenesis–and exogenous–cultured stem cells–sources). The progressive, marvelous results of such research (exceeding now 9.500 scientific papers in a PubMed search for “adult neurogenesis”) undoubtedly led to deep knowledge of the cellular and molecular mechanisms regulating the neural stem cell biology [reviewed in (31, 32)]. In parallel, studies carried out on mammalian species different from mice and rats started to show that adult neurogenesis occurrence, extension, rate, behavioral role(s), and function(s) can be heterogeneous among mammals [reviewed in (8, 33, 34) Figure 1)]]. Beside striking neurogenic processes in unconventional regions of only some animal species [e.g., the striatum and cerebellum in rabbits; (35–38)], even the apparently conserved hippocampal neurogenesis revealed remarkable differences among mammals, such as in bats [very low levels and high species-specific differences in its rate; (39)] or in dolphins [absence of hippocampal neurogenesis; (40)]. Wild mammals show species-specific, rather stable hippocampal neurogenesis, the rapid adaptation of hippocampal neurogenesis to experimental challenges being a characteristic of laboratory rodents (41).

Most importantly, adult neurogenesis appears to be highly reduced in humans with respect to rodents: while in mice and rats the genesis of new neurons is a lifelong process, though declining with age, in humans the forebrain and hippocampal stem cell niches are not more detectable at 2 and 7–13 years of age, respectively (42–44). Recent work carried out in dolphins, namely, mammals devoid of olfaction, and brain olfactory structures, showed that no periventricular neurogenesis is detectable after birth in these large-brained, long-living mammals (40, 45). Taken together, the results obtained in human and dolphins strengthen the view that neurogenic processes in mammals are strictly linked to specific physiological functions (e.g., olfaction) rather than to brain repair [see (46)].

## The “immature neuron” story

**Figure 2:**
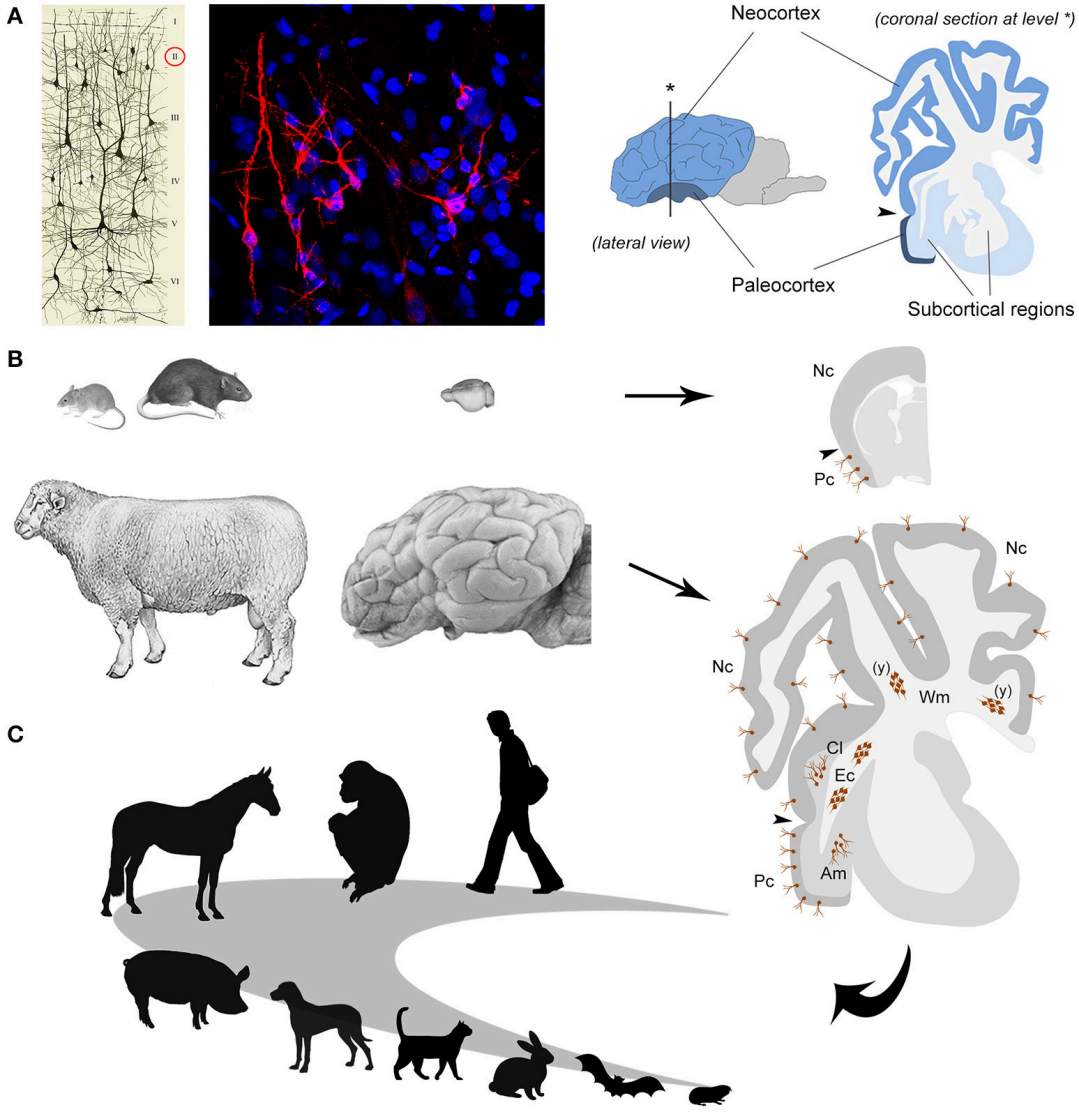
The immature neurons as an example of comparative medicine. **(A)** left, immature neurons revealed in the cerebral cortex layer II by immunocytochemical detection of the cytoskeletal protein Doublecortin (in red; blue, nuclei counterstained with DAPI); right, localization and extension of paleo- and neo-cortex in the mammalian brain [drawing reproduced from (50)]. **(B)** Laboratory rodents and sheep differ in their brain size and gyrencephaly (left and middle); the occurrence of immature neurons (brown cells, both single and forming clusters) is restricted to the paleocortex in the former whereas it extends to the neocortex and some subcortical and white matter regions in the latter (right); Pc, paleocortex; Nc, neocortex; Am, amygdala; Cl, claustrum; Ec, external capsule; Wm, periventricular white matter; (y) only at postnatal/young ages. **(C)** Future perspectives: studies of comparative medicine can help to understand the logic of evolutionary adaptation of the immature neurons (their occurrence, extension, and relative amount) in different mammalian orders and species, including humans.

In the nineties, in parallel with the first studies on adult neurogenesis, another population of cells which continue to express markers of immaturity throughout life (i.e., doublecortin–DCX–and polysialylated Neural Cell Adhesion Molecule–PSA-NCAM) was discovered in the cortical layer II of the rodent piriform cortex (47–49) (Figure 2A). Later, these cells were called “immature” neurons (51), since they are generated prenatally and considered as a potential reservoir of young neurons which might progressively mature to be integrated into circuits of the adult cerebral cortex at different ages, in a sort of “delayed neurogenesis” [hypotheses developed in (52, 53)]. Recently, by using a tamoxifen-inducible transgenic mouse model in which DCX-expressing cells can be permanently labeled with a green fluorescent protein, it was shown that most cortical immature neurons of the mouse piriform cortex actually mature across ages, being no more visible after ceasing to express the immaturity markers (54). Although analyses conducted with different cell and synaptic markers strongly suggest that some of these neurons become structurally integrated [(54, 55), in sheep], a clear and definitive proof that they can function into mature neural circuits is still lacking.

If further studies are needed to go deeper in the molecular mechanisms regulating the mature/immature states of the cells, other urgent questions are open on the side of comparative medicine, since we know from preliminary data that the topographical distribution of immature neurons can be different in some mammalian species. Whereas in mice and rats they are confined to the evolutionarily ancient cortex (paleocortex), in rabbit, guinea pig, some bat and non-human primate species, they have been found to extend into the neocortex (56–61). The comparative studies seem to reveal a turning point in mammalian brain plasticity, suggesting that immature neurons might be far less abundant in the species relying on high rates of lifelong neurogenesis, such as the laboratory rodents.

## Immature neurons in sheep: the importance of comparative neuroscience

Sheep are mammals showing some features closer to humans than to rodents, such as relatively long lifespan [15–20 years] and larger, gyrecefalic brain [weight: 118–140 grams; gyrification index: 1.94–2.29; (62)]. At the same time, it is still possible to perform experiments usually carried out on mice and rats, including BrdU treatment and brain tissue perfusion, allowing to study neuronal plasticity at a cellular level in physiological and experimental conditions (55, 63). On these bases, we recently investigated the occurrence and anatomical distribution of immature neurons in adult sheep, showing that they are surprisingly abundant in this large-brained mammal with respect to rodents (55). Of importance, they not only extend into the whole neocortex but can also be found in several subcortical regions, including important gray matter nuclei (amygdala and claustrum, involved in emotion-related memories and consciousness) and white matter bundles [(55); Figure 2B)]. The BrdU treatment (performed both during pregnancy and adulthood) allowed to prove that the DCX+ cells in all those regions were generated prenatally and do not divide in the adult, thus being actually immature neurons. These data support the hypothesis that immature neurons might be more represented in animals with relatively large and “complex” brains with respect to rodents, unlike neurogenic processes which become quite reduced in large-brained, long-living species (8, 64, 65). Furthermore, the sheep study establishes that immature neurons must not be considered restricted to the cerebral cortex layer II, implying that studying the other, subcortical cell populations will require non-rodent animal models.

Among the novel findings emerging from the sheep brain, were also large clusters of doublecortin-positive, non-newly generated cells in the dorsal (periventricular) white matter of very young animals (see Figure 2B). The same structures, reminiscent of “trapped” immature neurons, were present in neonatal dolphins (grouped with sheep in the order Cetartiodactyla), then disappearing in both species around puberty (66). This is quite different from what is known to occur in early postnatal brains of rodents and humans, characterized by the migration of isolated neuroblasts toward the cortex. Again, a remarkable heterogeneity of structural plasticity is confirmed in the brain of mammals, here explained by the different stages of development at birth (highly advanced in Cetartiodactyla with respect to rodents and humans).

Hence, studies carried out on non-rodent species reveal that large-brained mammals harbor different types of brain plasticity, especially considering the topographical distribution and amount of the “whole cell” plasticity. On these bases, laboratory rodents (while remaining a good model for molecular and cellular mechanisms) cannot be the right model for studying the outcome of some processes, such as adult neurogenesis and immature neurons, since they are not present in some brain regions and/or occur at different rates in small- and large brained mammals [see (65) for evolutionary hypotheses]. For instance, sheep might be used to investigate behaviorally/environmentally-induced modulation of immature neurons in neocortical areas, amygdala and claustrum.

## Future perspectives

Dealing with brain plasticity, the alternative scenarios opened by comparative results with respect to most research conducted on laboratory rodents strongly indicates a need for comparative medicine involving a wide range of animal species, including those of veterinary interest. It is naïve to think that after unraveling the cellular and molecular mechanisms of neural plasticity and brain stem cells in rodent models, the same outcome in terms of behavior and outcome of their progeny, can be simply obtained in brains which, through million years, have evolved different neuroanatomy, size, regional complexity, functions [see (2, 9)], and in which even the putative existence/role(s) of different types of “natural” plasticity appear to be different (8). The dramatic drop in adult neurogenesis in some long-living mammals is a prominent example for the need for a wider “phylogenetic” view in order to fill the gap between mice and men (64, 65).

Accordingly, the emerging opportunity of immature neurons should be investigated further on by considering a wide range of mammals, including humans (Figure 2C). We still lack systematic quantitative, really comparable data on the distribution of these neurons in mammalian species which widely differ in their neuroanatomy (brain size, gyrencephaly, cortical extension) and other internal/external features (lifespan, ecological niche, domestication vs. wildness, food habit). The question is: are the relative amount and topographical distribution of immature neurons homogeneous across mammals? If not: is there a trend/relationship with some of the above-mentioned anatomical/behavioral features? Moreover, it could be something major at stake. Some studies performed in sheep and macaque monkeys to determine the time course of adult neurogenesis in the olfactory bulb (sheep) and dentate gyrus of the hippocampus (both species), showed that neuroblast maturation is protracted over a minimum of a 3–6 months, respectively, thus resulting delayed several times longer than in rodents (67, 68). These data are in accord with different rates of cell proliferation markers (e.g., Ki-67 antigen) vs. immaturity markers (e.g., doublecortin) detection in rodent and non-rodent species, with a higher persistence of the immaturity markers in the latter [reviewed and discussed in (8)]. Taken together, these observations suggest that long time maturation and accumulation of “immature” neurons might be a feature of long-living species, even within the neurogenic sites. Yet, also in this case, the data available are still fragmentary and mostly qualitative. Only accurate and comparable quantitative analyses across different species could help to establish if this trend can be confirmed, and, in that case, sheep can be useful animal models to unravel the mechanisms of such delayed maturation (as medium-sized domestic animals, they can be employed in behavioral tests and subsequent non-invasive imaging techniques; (69, 70). Understanding whether the young neurons (both newlyborn and generated pre-natally but “immature”) can mature differently in widely different mammals might be of paramount importance for translational perspectives.

In conclusion, brain structural “whole cell” plasticity is a typical example for the need of comparative medicine in order to unravel the evolutionary aspects determining different outcomes of the neurogenic/other plastic processes in different mammals, thus avoiding to sleep into biases when figuring out translational outcomes based exclusively on the use of laboratory rodents.

## Author contributions

All authors listed have made a substantial, direct and intellectual contribution to the work, and approved it for publication.

### Conflict of interest statement

The authors declare that the research was conducted in the absence of any commercial or financial relationships that could be construed as a potential conflict of interest.
